# Classic congenital adrenal hyperplasia: A delayed presentation

**DOI:** 10.12669/pjms.291.2830

**Published:** 2013

**Authors:** Saima Aziz Siddiqui, Nargis Soomro, Ashraf Ganatra

**Affiliations:** 1 Dr. Saima Aziz Siddiqui, MBBS, MCPS, FCPS, Assistant Professor, Department of Obstetrics & Gynaecology, Dow University of Health Sciences & Civil Hospital Karachi, Karachi, Pakistan.; 2 Dr. Nargis Soomro, MBBS, DA, FCPS, FRCOG, Professor, Department of Obstetrics & Gynaecology, Dow University of Health Sciences & Civil Hospital Karachi, Karachi, Pakistan.; 3 Dr. Ashraf Ganatra, MBBS, M.S(Plastic Surgery), Professor, Department of Plastic Surgery, Dow University of Health Sciences & Civil Hospital Karachi, Karachi, Pakistan.

**Keywords:** Congenital adrenal hyperplasia, Clitoromegaly, Virilization, Clitoroplasty, Ambiguous genitalia

## Abstract

Congenital adrenal hyperplasia (CAH) is a rare congenital disorder, which in cases of female genotype may result in virilization. Specific enzyme deficiencies in adrenocorticoid hormones biosynthetic pathway lead to excess androgen production causing virilization. Classic type presents early in infant life as salt losing or simple virilizing type, whereas non classic form presents late at puberty or in adult life. Depending on the type of classic CAH, type of adrenocorticoid deficiency, extent of virilization & genotype, surgical corrective procedures, glucocorticoid & mineralocorticoid replacement therapy are the mainstay of management. We present here a case of classic congenital adrenal hyperplasia of simple virilizing type, which presented later in childhood.

## Introduction

 Birth of neonate is immediately accompanied by assignment of gender based on features of external genitalia. Gender assignment becomes a dilemma if external genitalia do not conform to the normal male or female appearance. Ambiguous genitalia, the congenital condition in which the external genitalia do not conform to either sex, give rise to extreme anxiety & concern to the parents. 

 Early but accurate & appropriate diagnostic evaluation is required in such cases. Key feature in physical examination is the presence of gonads in labioscrotal folds or inguinal canal. In the absence of palpable gonads, possibility of female pseudohermaphrodite is considered whereas in case of palpable gonads diagnostic evaluation is directed to male pseudohermaphrodite. 

 Among the variety of causes of ambiguous genitalia, congenital adrenal hyperplasia (CAH) is the commonest reported cause in the presence of 46XX karyotype.^1^^,^^2^ Virilized females with 46 XX karyotype have ambiguous external genitalia but normal internal female genitalia. During first seven weeks of gestation external genitalia of male & female genotypes are identical. In the absence of testosterone & dihydrotestosterone (DHT) external genitalia appear phenotypically female. DHT, the converted form of testosterone under the action of enzyme 5α reductase, acts on urogenital sinus & genital analgae to affect male differentiation of external genitalia. 5 α reductase is present in urogenital analgae in both male & female fetuses. Exposure of abnormal androgen levels cause masculinization of female external genitalia. Absence of antimullerian hormone leads to normal female internal genital organs. Any posterior labial fusion (defined as a ratio of the distance from anus to fourchette/anus to base of clitoris >0.5) or clitoromegaly (>1cm clitoral length) constitutes ambiguous genitalia and is indicative of a virilizing process.

## Case Report

 We report here a case of ambiguous genitalia resulting from congenital adrenal hyperplasia presenting later in childhood. 

 A 7 & 1/2 years female child was brought in outpatients clinic with history of ambiguous genitalia since birth. She had five siblings including an elder sister. Mother’s history did not reveal intake of antiepileptic drugs, progestogens or other drug treatment. There was no history of genital ambiguity in parents or any of the family members. Child was noted to have abnormal genitalia at the time of her birth at home, but medical help was never sought for that. She was in a good state of health with weight 30 kg & height 137 cm. Blood pressure was 100/60 mm of Hg.

 Examination revealed no breast development, no axillary hair. On genital examination there were a few pubic hairs, clitoromegaly i.e. stretched phallus size 5cm, labia majora & minora normally developed no fusion abnormality, normal introitus, gonads not palpable, single uretheral opening below the base of phallus. These findings were consistent with Prader stage 1 for ambiguous genitalia.^3^^,^^4^ There was no abnormality on systemic examination. On laboratory investigations, serum Na was 143meq/l, potassium 4meq/l.

 Ultrasound pelvis revealed uterus normal size for age, 3.5 cm in length & 2.1cm in transverse diameter, central midline echo, right ovary 2.3 X1.4 cm, left ovary 2.1 X1.4cm. karyotype was 46XX. 

 On the basis of raised 17αhydroxyprogesterone, testosterone, & Dehydraepiandrostanedione Sulphate (DHEAS) & decreased cortisol response to Adrenocortictropic Hormone (ACTH) stimulation in these results, alongwith normal karyotype & clitoromegaly in the presence of normal introitus, labia & female reproductive organs, classic congenital adrenal hyperplasia due to 21 hydroxylase deficiency was diagnosed. Her management comprised surgical correction of enlarged phallus by clitoroplasty with dorsal neurovascular bundle preservation & glucocorticoid replacement therapy. The operation was performed by the team of plastic surgeon & Gynaecologist.

 A traction suture of 3/0 poplygalactin, was placed in the glans of clitoris. Circumferential incision was given over base of the glans. Two longitudinal incisions were made lateral to dorsal neurovascular bundle. Corpra cavernosa visualized in entire extent. Excision of a portion of corpoeal tissue with wedge resection of glans was done, preserving neurovascular bundle. Conserved portion of corpora folded & sutured to pubic symphisis. Reconstruction of skin of labia minora & hood of clitoris was done.

 Postoperatively she recovered well with no complications. She was also started on replacement therapy with hydrocortisone.

**Fig.1:**
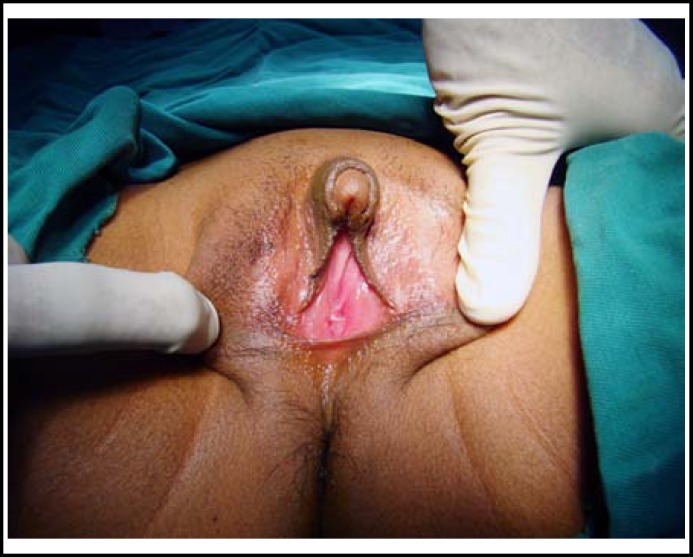
Clitoromegaly with normal labia & introitus.

## Discussion

 Incidence of CAH is reported as 1 in 15000 to 16000 births. These are a group of monogenic autosomal recessive disorders characterized by impaired cortisol synthesis & abnormal adrenal hormonal profile. These disorders are caused by deficiency of enzymes necessary for the synthesis of adrenal corticosteroids. Spectrum of severity of this condition ranges from severe salt losing type due to aldosterone & cortisol deficiency to a milder form with mild androgen excess. Important steps in the biosynthesis of corticosteroids involve conversion of pregnenolone & 17 hydroxy pregnenolone to progesterone & 17 hydroxy progesterone respectively, mediated by enzyme 3 β hydroxyl steroid dehydrogenase (3β HSD). These compounds in turn are converted to Deoxycorticosterone (precursor of aldosterone) & 11 deoxycorisol through the action of 21α hydroxylase [21α OHD] Final conversion of each of these precursors to Corticosterone & cortisol respectively is mediated through 11 β Hydroxylase [11β OHD]. Enzymes necessary for these steps in biosynthesis, if deficient lead to diversion of this pathway away from corticosterone & Cortisol synthesis to androstanedione & testosterone synthesis. Cortisol is the only adrenal hormone able to stimulate biofeedback loop. Corticosteroids deficiency leads to raised adrenocorticotrophic hormone (ACTH) which stimulates adrenal cortex to produce more androgenic hormones leading to features of masculinization (development of male characteristics in a genotypical female) of female fetus resulting in ambiguous genitalia. Thus deficiency of any of the three mentioned enzymes may lead to congenital adrenal hyperplasia leading to virilization. Most common enzyme deficiency in causing CAH is 21α OHD, accounting for 90% cases of CAH.^5^ Whereas 11β OHD accounts for 5-8% cases.^4^^,^^5^ 21α OHD is a cytochrome P450 enzyme located in endoplasmic reticulum. There are 2 genes encoding for 21 hydroxylase, the structural gene encoding p450c21 (CYP21) & pseudogene CYP21p, both located on chromosome 6p21.3.^4^ Incidence of 21α OH deficiency varies between 1 in 5000 to 1 in 15000 based on neonatal screening programmes.^5^ A hospital based study from Karachi reported diagnosis of congenital adrenal hyperplasia in 41.44% cases of ambiguous genitalia.^6^ It also showed that consanguinity of parents was identified in 52% of these cases.

**Fig.2:**
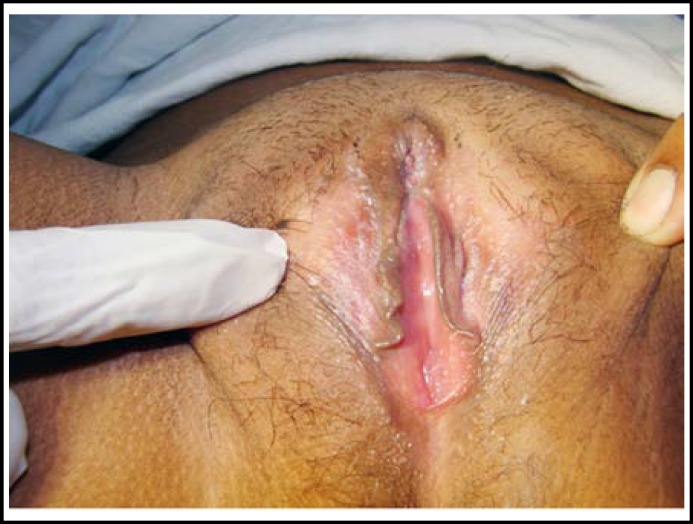
After one month of clitoroplasty.

 According to time of presentation of androgenization, CAH can be of classic or non classic type. The classic type, presents early in life as salt losing type or simple virilizing type, depending on presence of both cortisol & 11 deoxycorticosterone (DOC) deficiency or sole deficiency of cortisol. Affected females are born with enlarged clitoris, fused labioscrotal folds & a urogenital sinus which may become a phallic urethera. Affected females can present a range of virilization of external genitalia from mild clitoromegaly & posterior fusion to complete labioscrotal fusion & urogenital sinus which may open on perineum or shaft of phallus. 21α OHD deficiency leading to failure of conversion of progesterone to 11-deoxycorticosterone may result in aldosterone deficiency which is called “salt losing type” of CAH. It accounts for 75% cases of classic CAH, in which neonate develops dehydration, hypotension & hyponatremia between 7-28 days of life. In “non salt losing” type there is lesser degree of masculinization as compared to salt losing type,^3^ 11β OHD deficiency is the second commonest cause of classic CAH. It is the hypertensive form of CAH, as absence of 11β OHD leads to accumulation of 11 deoxycorticosterone. Though aldosterone levels are decreased but salt retaining properties of 11 deoxycorticosterone result in hypertension.

**Table-I:** Endocrinological assay results

*Investigation*	*Result*	*Reference range*
17αhydroxyprogesterone	100ng/ml	(0-0.6ng/ml)
Serum testosterone	103.7ng/dl	(For 7-12years age; 3-68ng/dl)
DHEAS	212.3µg/dl	(Prepubertal range19-63µg/dl)
Serum Aldosterone	13.3ng/dl;	(4-31ng/dl)
Short synacthen test	Baseline 3.4µg/dl	(>18µg/dl)
	30 minutes 4.8µg/dl	
	60 minutes 5.5µg/dl	

 Non classic type of CAH presents later in childhood or near puberty & is characterized by normal production of cortisol & aldosterone with excess production of androgens. It is always caused by 21α OHD & only rarely due to other causes. Non classic CAH is common with an incidence of 1 in 100 to 1 in 300.^4^^,^^5^

 Diagnosis of 21α OHD is made with raised serum 17 hydroxyprogesterone. In 11β OHD deficiency, 11 deoxycorticosterone level is raised whereas in 3β HSD deficiency, serum 17 α hydoxypregnenolone & DHEAS are raised.^7^

 Management of cases of ambiguous genitalia depends on location of gonads, adequacy of phallus, size & location of vaginal orifice; if present need to be assessed.^8^ Surgery is tailored according to amount of virilization to best suit the gender role. Early diagnosis & management are likely to yield best possible outcome. For clitoral reduction & recession major corrective surgery is ideally suitable at 3 to 6 months of age.^8^ Surgical techniques of preservation of neurovascular bundle & glans as well as corporeal preservation including recession dismembered clitoroplasty are increasingly being favoured.^8^^,^^9^ Our case was classic type of CAH but instead of infancy she presented late in childhood despite the abnormality being identified at birth. There are local & Indian reports of CAH presenting in later childhood despite being recognized at birth.^10^^,^^11^

## Conclusions

 Management of CAH is a complex issue due to delayed help seeking & lack of neonatal screening programmes in our set up. Accurate diagnosis of this rare disorder requires specialized endocrinological assays, which are not easily available at public sector set up for poor patients. Expert genital reconstructive surgery tailored to best suit the gender role are prerequisite for proper management so as to enable social & psychological adjustment of affected patients.
